# CD4+ and γδ T Cells are the main Producers of IL-22 and IL-17A in Lymphocytes from *Mycobacterium bovis*-infected Cattle

**DOI:** 10.1038/srep29990

**Published:** 2016-07-18

**Authors:** Sabine Steinbach, H. Martin Vordermeier, Gareth J. Jones

**Affiliations:** 1 TB Immunology and Vaccinology, Department of Bacteriology, Animal and Plant Health Agency, New Haw, Addlestone, Surrey, KT15 3NB, United Kingdom

## Abstract

Gene transcription studies have identified dual roles for the cytokines IL-17A and IL-22 in bovine tuberculosis, where they show potential as both predictors of vaccine success and correlates of infection. To allow for a detailed investigation of the cell populations responsible for production of these cytokines, we have utilised a novel bovine IL-22 specific recombinant antibody for flow cytometry. Bovine tuberculin (PPDB) induced greater IL-22 and IL-17A production in *Mycobacterium bovis (M. bovis)*-infected cattle compared to non-infected controls, while PWM-induced cytokine levels were similar between the two groups. In *M. bovis*-infected animals, PPDB specific IL-22 and IL-17A responses were observed in both CD4+ T cell and γδ T cell populations. Although both cytokines were detected in both cell types, IL-22/IL-17A double producers were rare and confined mainly to the γδ T cell population. These results support previous gene transcription studies and extend the observation of increased IL-22 and IL-17A responses in *M. bovis*-infected animals to the level of protein production. We were also able to characterise the cell populations responsible for these disease-related cytokine responses. The data generated can be used to further our understanding of the immunopathology of bovine tuberculosis and to produce more sensitive and specific immune-diagnostic reagents.

*Mycobacterium bovis (M. bovis*), the main causative agent of bovine TB and a member of the *M. tuberculosis* complex, is capable of causing disease in humans and infecting a wide array of wildlife species, including domestic cattle. Bovine TB represents a significant economic animal health problem, where it has been estimated that globally the disease costs US$3 billion annually^1^. In Great Britain, the primary screening test for diagnosing bovine TB in cattle is the single intradermal comparative cervical tuberculin skin test (SICCT). In addition, the whole blood interferon-γ (IFN-γ) release assay can be used as an ancillary test to the skin test, resulting in improved detection of *M. bovis-*infected cattle. However, despite the current ‘test and cull’ programme in Great Britain, the annual number of new herd breakdowns remains high in the West of England and in Wales, resulting in the slaughter of over 300,000 cattle in the decade up to 2013^2^. Thus, new control strategies are being investigated, including cattle vaccination and improved diagnosis using additional biomarkers of infection. It is hoped that by gaining a better understanding of immunity to *M. bovis,* correlates of immune protection or disease progression can be identified that would facilitate the design of effective TB vaccines, improved diagnostic and therapeutic strategies.

The last decade of research in pulmonary immunology has identified key molecules required for pathogen detection and clearance, with IL-17A and IL-22 emerging as major effector cytokines^3^. IL-17A is induced immediately after pulmonary *M. bovis* bacille Calmette-Guérin (BCG) infection of mice^4^ and contributes to the host’s immune defence by the induction of chemokines and cytokines responsible for the early recruitment of neutrophils and granuloma formation^4^,^5^. Recent reports have further suggested that this early IL-17A production is necessary for driving an effective Th1 immune response and robust IFN-γ production following BCG infection of mice^6^ and co-localisation of CXCR5+ T cells with *M. tuberculosis*-infected macrophages, an event crucial for optimal pathogen control^7^. IL-17A is also detected at later stages of *M. tuberculosis* infection in mice, suggesting an important role for Th17 cells in memory or secondary immune responses^8^,^9^,^10^. Although less well studied, a protective role for IL-22 has also been suggested. IL-22 from NK cells inhibits the intracellular growth of *M. tuberculosis* in human macrophages by enhancing phagolysosomal fusion^11^,^12^ while human NK cells producing IL-22 are required for BCG vaccine efficacy^12^. Indeed, in cattle vaccination/challenge experiments, higher levels of IL-17A^13^,^14^ and IL-22^15^ expression seen post vaccination but pre-challenge were positively associated with vaccine success (i.e. prevention of pathology) following subsequent challenge with *M. bovis*.

Although IL-17A production plays a role in host defence against mycobacterial infections, excessive production may contribute to immune pathology. Repeated exposure of *M. tuberculosis*-infected mice to BCG resulted in increased IL-17A expression, influx of neutrophils and lung tissue damage, which was abrogated in the presence of IL-17A-blocking antibodies^16^. Furthermore, greater IL-17A expression was observed in peripheral blood mononuclear cells (PBMC) from *M. bovis*-infected cattle that developed macroscopic lesions^17^, and positive associations between mycobacterial antigen-induced IL-17A production and lesion severity or mycobacterial burden have been demonstrated in cattle experimentally infected with *M. bovis*^18^. Indeed, our own gene transcription studies demonstrated increased IL-17A and IL-22 mRNA expression in *M. bovis*-infected cattle following stimulation with PPDB, but not in un-infected controls^19^, suggesting that both cytokines may be useful biomarkers for infection.

In most of the cattle studies detailed above, IL-17A and IL-22 responses were measured from a PBMC population, and the precise nature of the cells responsible for producing these cytokines were undefined. Given that both cytokines have been implicated as biomarkers for two very different applications: (i) predictors of vaccine success and (ii) correlates of infection, it is possible that different cell types may be active in these settings. However, to allow for such future investigations, methodologies must first be established that allow detailed characterisation of these cellular immune responses to *M. bovis* antigens. To this end, we have utilised a novel bovine IL-22 specific recombinant antibody for use in intracellular flow cytometry, which revealed both CD4+ T cells and γδ T cells as the major producers of IL-17A and IL-22 in the setting of bovine TB.

## Results

**Figure 1:**
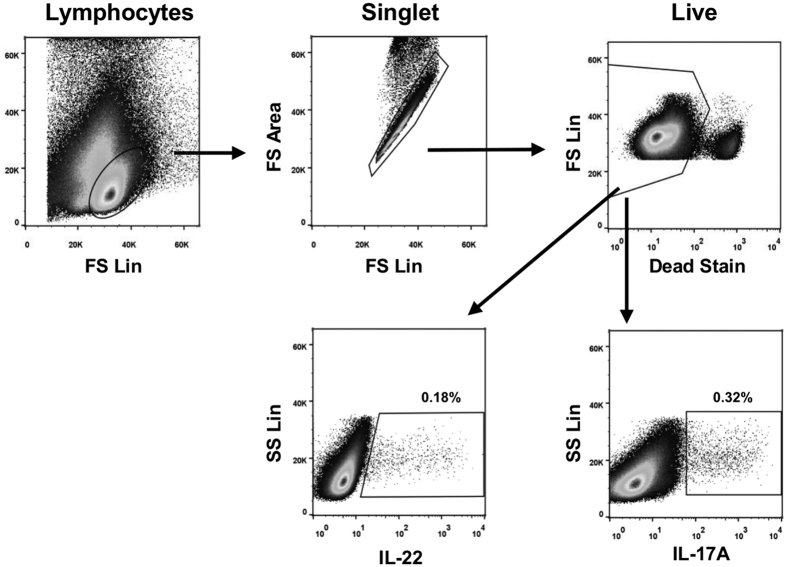
Identification of IL-22 and IL-17A production in bovine lymphocytes. PBMC from a TB-reactor animal were stimulated with PPDB and flow cytometry analysis performed to enumerate IL-22 and IL-17A production. Numbers represent the percentage of single, live bovine lymphocytes producing each cytokine.

**Figure 2:**
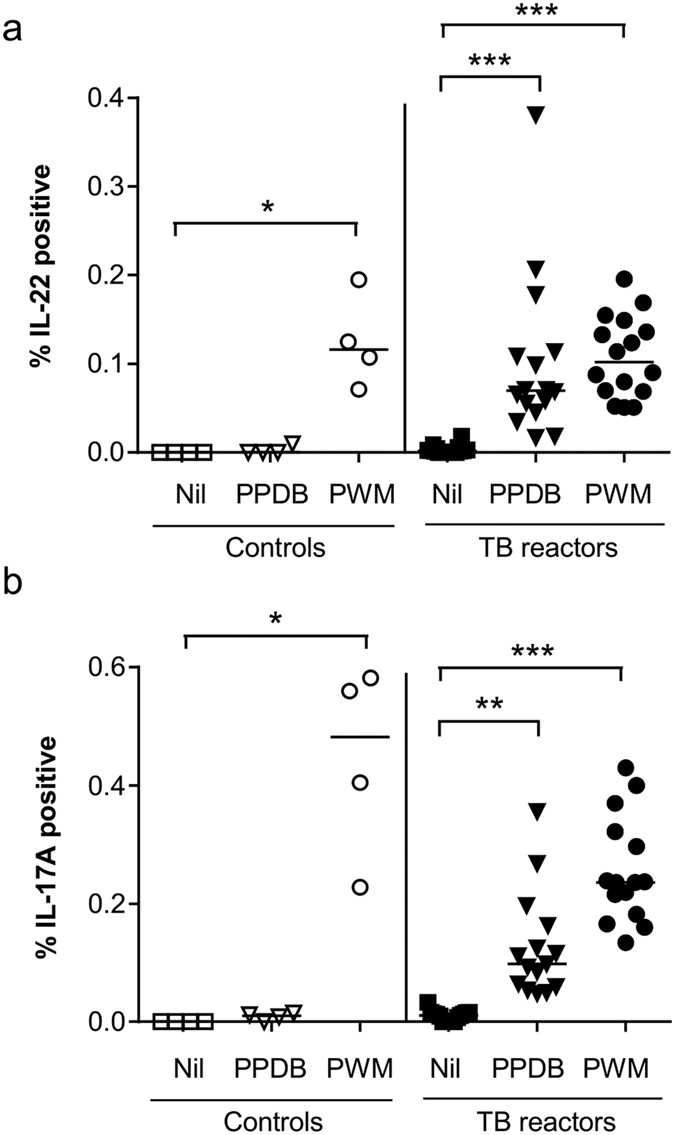
*M. bovis*-specific induction of IL-22 and IL-17A protein production. The percentage of bovine lymphocytes labelling positive for (**a**) IL-22 and (**b**) IL-17A protein in *M. bovis*-infected (TB reactors) and non-infected (control) animals. Each symbol represents an individual animal while horizontal lines represent the median value. *p < 0.05, **p < 0.01, ***p < 0.001, nonparametric ANOVA with Dunn’s Multiple Comparison Test.

To characterise the cellular components that respond to stimulation with mycobacterial antigens by producing IL-17A and/or IL-22, we developed multiparameter flow cytometry panels. The gating strategy used is shown in Fig. 1, which clearly demonstrates the ability of our system to identify and enumerate bovine lymphocytes producing IL-22 and IL-17A in response to stimulation with PPDB. These experiments were repeated in a larger number of cattle naturally infected with *M. bovis* (TB reactors) as well as uninfected control animals to enumerate the percentage of lymphocytes producing either IL-22 (Fig. 2a) or IL-17A (Fig. 2b). Compared to unstimulated cultures (Nil), mitogen (PWM) stimulation of PBMC from either non-infected control animals or *M. bovis*-infected TB reactors resulted in a greater percentage of IL-22 positive lymphocytes, confirming the capacity of cattle lymphocytes to produce IL-22 (Fig. 2a). Further, a significant increase in the percentage of IL-22 producing lymphocytes was observed in *M. bovis*-infected TB reactors following stimulation with PPDB. In contrast, no similar increase was observed in control animals following PPDB stimulation, demonstrating that IL-22 responses were specific for *M. bovis* infection. Similar results were also seen for IL-17A responses (Fig. 2b). Whereas mitogen stimulation induced significant increases in the percentage of IL-17A producing lymphocytes in both control and *M. bovis*-infected animals, *M. bovis* antigen stimulation only induced significant responses in *M. bovis*-infected animals.

**Figure 3:**
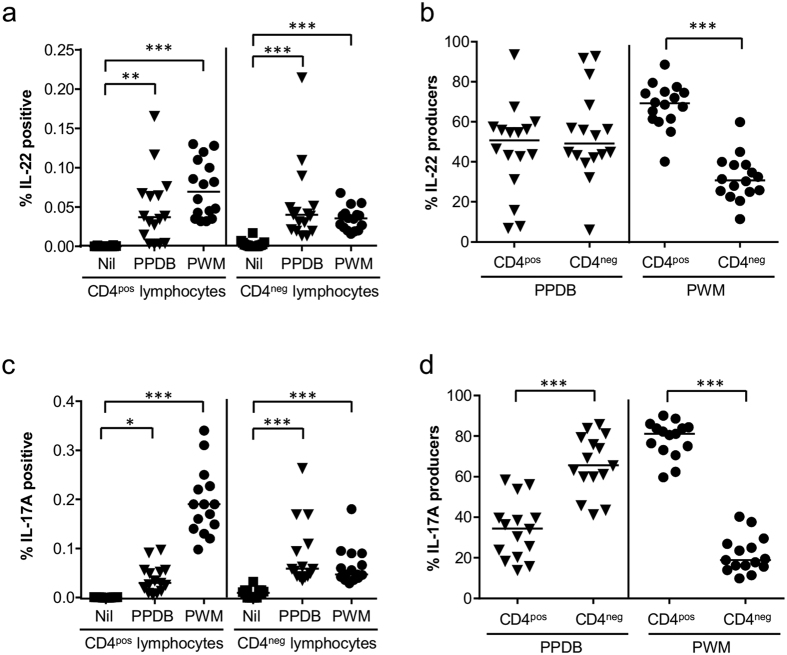
*M. bovis*-specific induction of IL-22 and IL-17A protein production in both CD4^pos^ and CD4^neg^ lymphocyte populations. (**a,c**) The percentage of bovine CD4^pos^ or CD4^neg^ lymphocytes from *M. bovis*-infected animals that label positive for (**a**) IL-22 and (**c**) IL-17A production. (**b,d**) The relative proportion of (**b**) IL-22 producers or (**d**) IL-17A producers that are either CD4^pos^ or CD4^neg^. Each symbol represents an individual animal while horizontal lines represent the median value. (a and c) *p < 0.05, **p < 0.01, ***p < 0.001, nonparametric ANOVA with Dunn’s Multiple Comparison Test; (**b,d**) ***p < 0.001 Paired Students T test.

Having shown that *M. bovis* antigens induced specific IL-22 and IL-17A responses in PBMC from *M. bovis*-infected cattle, we next identified the cell population responsible for this response. Mitogen stimulation induced significant increases in the percentage of IL-22 producing cells in both CD4^pos^ and CD4^neg^ lymphocyte populations (Fig. 3a). However, a significantly greater proportion of the IL-22 producing cells were found within the CD4^pos^ compared to the CD4^neg^ lymphocyte population (Fig. 3b). Similar to mitogen induced responses, *M. bovis* antigens induced significant increases in the percentage of IL-22 producing cells in both CD4^pos^ and CD4^neg^ lymphocyte populations. However, in contrast to mitogen stimulation, no clear dominance of either a CD4^pos^ or CD4^neg^ lymphocyte response was observed following antigen-specific stimulation, with responses showing a high degree of animal to animal variability. Similar response profiles were observed for IL-17A. Mitogen stimulation induced significant increases in IL-17A production in both CD4^pos^ and CD4^neg^ lymphocyte populations (Fig. 3c), but this again was dominated by responses in the CD4^pos^ lymphocyte population (Fig. 3d). *M. bovis* antigens induced significant increases in the percentage of IL-17A producing cells in both CD4^pos^ and CD4^neg^ lymphocyte populations. However, in contrast to IL-22, a significantly greater proportion of the IL-17A producing cells were located within the CD4^neg^ lymphocyte population.

**Figure 4:**
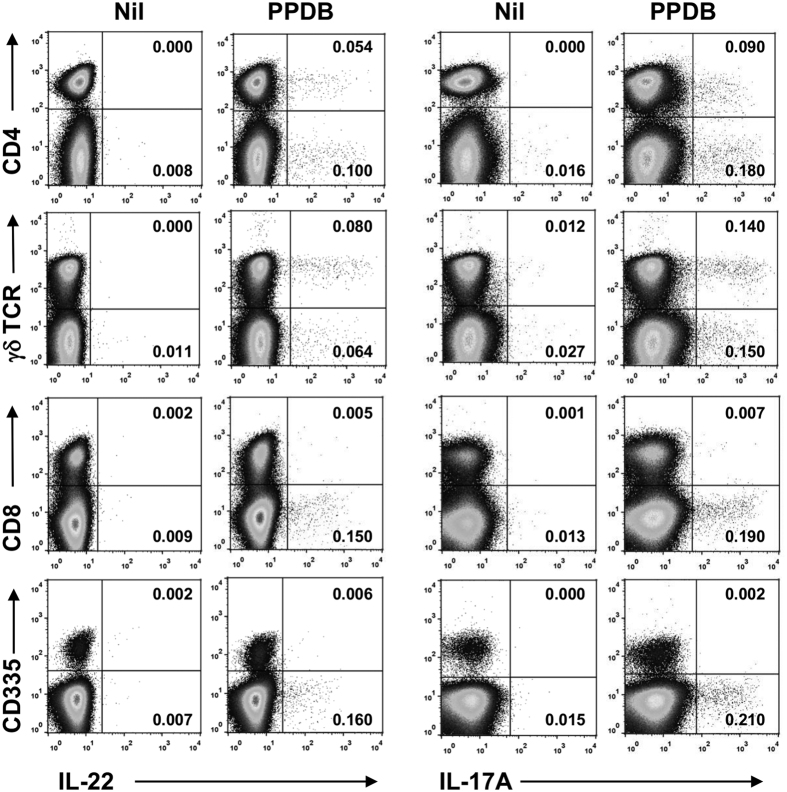
*M. bovis*-specific induction of IL-22 and IL-17A protein production in γδ T cells but not CD8^pos^ lymphocytes or NK cells. PBMC from a TB-reactor animal were stimulated with PPDB and flow cytometry analysis performed to enumerate IL-22 and IL-17A production in different lymphocyte populations. Numbers represent the percentage of single, live bovine lymphocytes. Data representative of at least 4 independent experiments.

To further define the cellular source of IL-22 and IL-17A, bovine lymphocyte populations were labelled with antibodies to CD4, CD8, CD335 and γδ TCR to identify CD4^pos^ T cells, CD8^pos^ T cells, NK cells and γδ T cells respectively. Representative results are shown in Fig. 4, where PPDB stimulation induced IL-22 production in both CD4^pos^ and CD4^neg^ lymphocyte populations (upper row). In addition, IL-22 production was also seen in both γδ TCR^pos^ and γδ TCR^neg^ populations (2^nd^ row). In contrast, IL-22 production could not be detected in CD8^pos^ (3^rd^ row) or in CD335^pos^ (bottom row) lymphocyte populations. Similar response profiles were also observed for IL-17A, where neither CD8^pos^ nor CD335^pos^ lymphocytes produced IL-17A following stimulation with mycobacterial antigens. Even in the presence of a strong non-specific stimulus such as PMA/Ionomycin, no IL-22 and IL-17A production were seen in the NK cell population, and only traces of these cytokines could be detected in CD8+ T cells (data not shown). The lack of IL-22 and IL-17A production in the NK cell and CD8+ T cell populations was not due to an inability of these cells to respond to mycobacterial antigens, as PPDB stimulation induced IFN-γ production in these cells (Supplemental Fig. 1).

**Figure 5:**
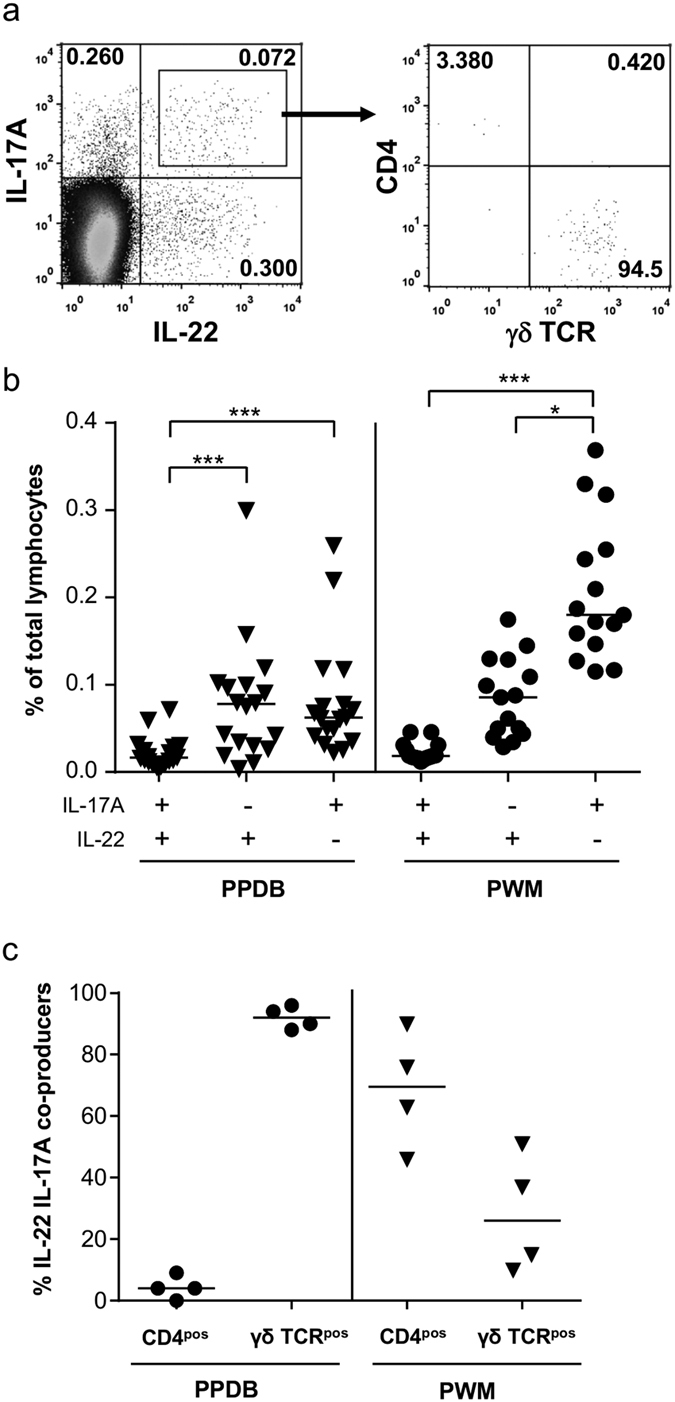
A small proportion of IL-22 producing lymphocytes co-produce IL-17A. (**a**) PBMC from a TB-reactor animal were stimulated with PPDB and flow cytometry analysis performed to enumerate simultaneous production of IL-22 and IL-17A in CD4^pos^ lymphocytes and γδ T cells. Numbers represent the percentage of single, live bovine lymphocytes in each quadrant (left hand panel) or the percentage of IL-22 IL-17A co-producers expressing CD4 or γδ TCR (right hand panel). (**b**) The percentage of lymphocytes producing IL-22, IL-17A or both following stimulation with PPDB or PWM. Each symbol represents an individual animal while horizontal lines represent the median value. *p < 0.05, ***p < 0.001, nonparametric ANOVA with Dunn’s Multiple Comparison Test. (**c**) The relative proportion of IL-22 IL-17A co-producers that label with either anti-CD4 or anti-γδ TCR. Each symbol represents an individual animal while horizontal lines represent the median value.

Given that similar response profiles were observed for IL-22 and IL-17A production, we investigated whether these two cytokines were being co-expressed by the same cell. Therefore, flow cytometry was used to identify bovine lymphocytes simultaneously producing IL-22 and IL-17A following stimulation with either *M. bovis* antigens (Fig. 5a, left hand panel) or mitogen (data not shown). The percentage of bovine lymphocytes expressing IL-17A only (Fig. 5a left hand panel, upper left quadrant), IL-22 only (Fig. 5a left hand panel, lower right quadrant) or IL-17A and IL-22 (Fig. 5a left hand panel, upper right quadrant) are summarised in Fig. 5b. Following stimulation with mycobacterial antigens, significantly fewer lymphocytes co-produced IL-17A and IL-22 compared to those singularly producing IL-17A or IL-22. Although a similar trend was also observed following mitogen stimulation, the percentage of bovine lymphocytes producing IL-17A only was significantly greater than that for IL-22 only or IL-17A IL-22 co-producers. Similar observations were made with IFN-γ, where IL-22 IFN-γ co-producers or IL-17A IFN-γ co-producers were rarely detected (data not shown). Given that a minor population of IL-22 IL-17A co-producers could be detected, we lastly determined the cell surface phenotype responsible for this response. Bovine lymphocytes co-producing IL-17A and IL-22 were identified (Fig. 5a left hand panel, upper right quadrant) and ‘back-gated’ for cell surface expression of CD4 and γδ TCR (Fig. 5a, right hand panel). Following stimulation with mycobacterial antigens, the majority of bovine lymphocytes co-producing IL-22 and IL-17A were γδ T cells (Fig. 5c). In contrast, there was a trend for a greater proportion of IL-22 IL-17A co-producers in the CD4^pos^ T cell population following mitogen stimulation, however more animal to animal variability was observed. Although the role of CD4^pos^ and γδ T cells as co-producers was only directly investigated in 4 animals (Fig. 5c), analysis of data from other experiments with a greater number of animals confirmed that a CD4^neg^ cell population was the major co-producer of IL-22 and IL-17A following PPDB stimulation of cattle PBMC (Supplemental Fig. 2, left panel).

## Discussion

We have previously demonstrated that mycobacterial antigens induce increased gene transcription for the cytokines IL-22 and IL-17A in PBMC from *M. bovis*-infected cattle but not from uninfected controls^19^. The results of this present study not only confirms these findings at the level of protein expression, but also identifies both CD4+ and CD4- T lymphocytes as major cell populations within cattle PBMC responsible for the production of these cytokines in response to *M. bovis* antigens. Furthermore, within the CD4- lymphocyte population, we identified γδ T cells as the major source of IL-22 and IL-17A production. As expected, our flow cytometry analysis also confirmed that cattle PBMC rarely contained cells co-expressing CD4 and the γδ T cell receptor (data not shown), demonstrating that the cytokine-producing CD4+ T cells and γδ T cells described in this study represented discrete cell populations.

Although studies in mice and humans have shown that populations of both CD4+ T cells and γδ T cells have the capacity to produce IL-22 and IL-17A^20^,^21^, similar data for these cell populations in cattle is limited. Using qRT-PCR, Ma *et al*. showed increased expression of IL-22 mRNA levels in purified populations of both cell types following stimulation with different mitogens^22^. With more direct relevance to bovine TB, Aranday-Cortes *et al*. demonstrated that mycobacterial antigens induced IL-22 mRNA expression in a purified population of CD4+ T cells from *M. bovis* infected cattle^19^. However, in both studies, the analysis of IL-22 expression was limited to gene transcription analysis, and the impact of this on actual protein levels was not investigated due to the lack of suitable reagents detecting IL-22 protein. With the development of the flow cytometry methods detailed herein, we can extend the conclusions from the two earlier studies and show for the first time that both mitogen and *M. bovis* antigen stimulation of bovine cells results in elevated protein levels of IL-22 in both the CD4+ T cell and γδ T cell compartments. Furthermore, our results for IL-17A production are in agreement with previous studies in *M. bovis* infected animals, where mycobacterial antigens induced increased IL-17A mRNA^19^ and protein^23^ expression in purified populations of both CD4+ T cells and γδ T cells.

The demonstration that bovine γδ T cells are a major cell population in cattle PBMC producing IL-22 following stimulation with *M. bovis* antigens differs from our previous study, where PPDB stimulation induced no IL-22 mRNA expression in highly purified γδ T cells^19^. To investigate this discrepancy, we repeated the experiment by Aranday-Cortes *et al*., but this time used flow cytometry analysis to quantitate the production of IL-22 at the protein level. Although PPDB induced IL-22 in purified populations of CD4+ T cells, minimal amounts were detected in purified populations of γδ T cells stimulated in the presence of monocytes or CD1b+ dendritic cells to act as APC (data not shown). However, in the same experiment, PPDB stimulation of total PBMC from the same animal resulted in production of IL-22 by the γδ T cell component. Thus, our results investigating protein levels are consistent with the previous study^19^ measuring mRNA expression, and suggest that additional signals provided by other cells present in the PBMC population are required to enable γδ T cells to produce IL-22 in response to *M. bovis* antigens. One such candidate may be IL-2. Elloso *et al*.^24^ demonstrated that *in vitro* proliferation of purified human γδ T cells in response to malarial antigens required CD4+ T cell production of a cytokine that signaled through the IL-2R, whilst other studies in cattle showed that *in vitro* activation and proliferation of purified γδ T cells required two signals, one delivered through the presentation of antigen by APC and a second delivered by addition of exogenous IL-2^25^,^26^.

Previous works exploring the relative contribution of distinct cell populations to the overall IL-17A response during mycobacterial infections have been conflicting. Whereas data from both mice^4^,^5^,^10^ and human^27^ studies identified γδ T cells as the predominant source of IL-17A production, other groups have shown that in the human^28^,^29^,^30^,^31^ and cattle setting^23^ CD4+ T cells were the major source of IL-17A. Possible factors that may contribute to these discrepancies include the species studied (e.g. mice, human or cattle), the nature of mycobacterial infection (e.g. BCG, *M. tuberculosis* or *M. bovis*), the cell source (e.g. lung cells, splenocytes or peripheral blood cells), the activating agent (e.g. different mycobacterial antigens or mitogens), and the assay system employed (e.g. analysis of complex cell mixtures or purified cell populations). Our results demonstrate that distinct cell populations produce IL-22 and IL-17A, and that their relative contribution to the overall cytokine response was also influenced by several factors, including the nature of the antigen, the cytokine studied and animal variability (Fig. 3). While mitogen stimulation clearly resulted in a significantly greater proportion of IL-22 and IL-17A producers found within the CD4+ T cell population, this was not evident following stimulation with mycobacterial antigens. Indeed, a CD4 negative population, which we subsequently showed to be γδ T cells, was the dominant cell population producing IL-17A following stimulation with PPDB. In contrast, we did not identify a clear dominance for either the CD4+ or CD4 negative cell population in the IL-22 response, due to a high degree of animal variation in IL-22 production in *M. bovis*-infected cattle. The discovery that γδ T cells from *M. bovis*-infected cattle produced IL-22 following stimulation with mycobacterial antigens is in contrast to a similar study performed in human TB patients, where IL-22 was expressed exclusively in CD4+ T cells^31^. Although differences in the assay may be a contributing factor (i.e. Scriba *et al*. stimulated whole blood for 12 hours while in our study we stimulated PBMC for 24 hours), our results may also highlight more fundamental species differences in γδ T cell responses between bovine and human TB infection.

The demonstration that mycobacterial antigens induced both IL-17A and IL-22 gene transcription in the CD4+ T cell population from *M. bovis* infected cattle^19^ led us to speculate that their production would be derived from the previously described CD4+ T helper 17 (Th17) cells, a population that has been shown to produce both cytokines^32^. Although we did detect these cytokines in the CD4+ T cell compartment, cells co-expressing IL-17A and IL-22 were rarely detected. This may be an artefact of the assay conditions used: we analysed both cytokines at the same time whereas it is possible that subtle differences in the kinetics of IL-17A and IL-22 protein production exist for this cell population. Alternatively, although bovine CD4+ T cells may retain some features of mouse Th17 cells, such as IL-17A production and CCR6 and IL-23R expression^33^, our results suggest that in the setting of mycobacterial infection they do not exhibit the ability to produce IL-22. This appears to be more akin to the human setting, where it has been suggested that Th17 cells rarely produce IL-22^34^. CD4+ T cells expressing IL-22, but not IL-17A or IFN-γ, have been described in humans^35^,^36^ leading to the classification of a Th22 cell population. Thus, we propose that the IL-22 expressing CD4+ T cells identified in our study may represent an analogous bovine Th22 population. Our results demonstrating that the majority of bovine CD4+ T cells responding to mycobacterial antigens produce distinct non-overlapping IL-17A, IL-22 or IFN-γ responses are similar to that previously shown in healthy mycobacteria-exposed humans, where specific IL-17A and IL-22 producing CD4+ T cells were distinct from each other and from Th1 cytokine producing cells^31^.

Although less common, our results also demonstrate the presence of a minor population of bovine lymphocytes capable of co-producing both IL-17A and IL-22 following stimulation with mycobacterial antigens. Interestingly, these cells were predominately CD4- γδ TCR+. These results are similar to that observed in a human study, where γδ T cells capable of producing both IL-22 and IL-17A were detected, although they constituted a rare population within the γδ T cell repertoire (e.g. only 2.7% of IL-22 producing Vγ2Vδ2 T cells also produced IL-17A)^37^. There is increasing evidence that γδ T cells play an important role in the host defence against mycobacterial infections in cattle^38^. In contrast to humans and mice, γδ T cells are more abundant in ruminant species, representing 10–20% of circulating lymphocytes in adult cattle. Following *M. bovis* infection, both the frequency and activation state of circulating γδ T cells increase, suggesting an active response to infection^39^. Furthermore, γδ T cells are one of the first cell populations to accumulate at sites of *M. bovis* infection^40^, and studies in a xenochimeric SCID-bovine mouse model suggest that this presence is important for proper formation of the developing granuloma and host survival following *M. bovis* infection^41^. One possible mechanism by which γδ T cells may regulate granuloma formation is through the production of IL-17A. IL-17A deficient mice fail to develop mature lung granulomas following BCG infection^5^, and γδ T cells have been shown to be major producers of IL-17A in the lungs of BCG infected mice^10^. Furthermore, adoptive transfer of IL-17A-producing γδ T cells into IL-17A deficient mice allowed for formation of granulomas^5^. Given that our results show that bovine γδ T cells from *M. bovis*-infected cattle produce IL-17A and IL-22, we may speculate that these cells, as shown in the mouse model, may also play a similar role in mature granuloma formation during bovine TB. Indeed, a recent study demonstrated IL-17A and IL-22 expression with tuberculous lesions in cattle, particularly in early lesions^42^. However, as the same study observed little accumulation of γδ T cells in early stage granulomas, there is still further work required to fully explore this hypothesis. Furthermore, given that cattle exhibit numerous sub-populations of γδ T cells, work is also required in fully characterising the γδ T cells producing IL-17A and IL-22, either singularly or co-producing, during bovine TB infection.

In summary, we report for the first time the existence of IL-22 producing CD4+ T cell and IL-22 and/or IL-17A producing γδ T cell subsets in cattle and their involvement in the immune response to mycobacteria in bovine TB infection. Remarkable is the contribution of mycobacteria-specific γδ T cells as the predominant IL-17A producing cell population and the major cell population that co-produced IL-22 and IL-17A. The new boIL-22 antibody offers us now the possibility to more closely analyse the IL-22 response in *M. bovis* infected cattle, and to further understand the role of IL-22 in innate and adaptive immunity which could benefit vaccine research and therapies for chronic inflammatory diseases. For example, when analysed prior to infection, previous studies in cattle have implicated vaccine-induced IL-17A^13^,^14^ and IL-22^15^ production as biomarkers capable of predicting vaccine success following subsequent infection with *M. bovis*. Identification of such biomarkers has potential impact on the screening of new bovine TB vaccines, allowing for initial gating criteria to be used when analysing vaccine-induced immunity that prioritises vaccines driving IL-17A and IL-22 responses for further evaluation in more costly vaccine challenge experiments. As we have shown that distinct cell populations are capable of producing these cytokines, further studies will be required to re-assess which of these responses are induced following vaccination and whether these particular cell populations mediate the association between IL-17A or IL-22 cytokine production and vaccine success. If indeed identified, this may lead the way to the rational design of bovine TB vaccines preferentially targeting these important cell populations.

## Methods

### Cattle

All animals were housed at the Animal and Plant Health Agency at the time of blood sampling, and procedures were conducted within the limits of a United Kingdom Home office license under the Animal (Scientific Procedures) Act 1986, which were approved by the APHA Animal Welfare and Ethical Review Body (AWERB) committee. Heparinised blood samples were obtained from naturally *M. bovis*-infected, SICCT (single intradermal cervical comparative tuberculin skin-test) -positive reactors from herds known to have bovine tuberculosis. All cattle were also positive in the whole blood IFN-γ assay described elsewhere^43^. Non-infected control cattle were obtained from TB-free herds located in non-endemic areas.

### Generation of recombinant boIL-22 monoclonal antibodies

Human recombinant monoclonal bivalent Fab antibody fragments were generated using the HuCAL (Human Combinatorial Antibody Library) technology (Bio-Rad AbD Serotec, Germany). The HuCAL library of Fab antibodies was screened with the recombinant full length bovine IL-22 protein (bovine IL-22 ORF minus signal sequence; aa 34–190^22^) which was expressed as a Fc-fusion protein in the mammalian cell line HKB11 (Bio-Rad AbD Serotec, Germany). Bovine IL-22 specific antibody clones were sequenced to identify unique antibodies for expression and purification. For our recombinant bivalent boIL-22 antibodies we chose the Fab-dHLX-MH format. These are bivalent Fab antibodies containing a heavy chain C-terminal dHLX-dimerisation domain followed by c-myc- and His-6-tag. Thirteen recombinant monoclonal antibodies with specificity for recombinant bovine IL-22 were generated. These antibody clones were investigated for their ability to detect IL-22 in PBMC of TB-reactor cattle stimulated with PPDB or PWM using intracellular flow cytometry. Ten of the IL-22 antibody clones detected IL-22 responses in bovine lymphocytes following stimulation with PPDB or PWM (data not shown). The specificity of the antibody clones for both recombinant and naturally produced bovine IL-22 were confirmed by use of these antibodies as capture antibodies in an IL-22 sandwich ELISA (data not shown). Antibody clone 58 was selected for all future experiments based on the intensity of labelling and the percentage of IL-22-positive lymphocytes detected.

### PBMC isolation and culture

PBMC were isolated from heparinised cattle blood by density gradient centrifugation using Histopaque-1077 (Sigma-Aldrich, UK). Purified PBMC were re-suspended in complete cell culture medium (RPMI 1640 containing 2 mM GlutaMax, 25 mM HEPES, 0.1 mM NEAA, 5 × 10^−5^ M β-mercaptoethanol, 100 U/ml penicillin, 100 μg/ml streptomycin [all from Gibco Life Technologies, UK] and 10% fetal calf serum [Sigma-Aldrich, UK]) and cultured at a concentration of 2 × 10^6^ cell/ml in the presence of bovine tuberculin purified protein derivative (PPDB, 1:100 dilution; Prionics, Switzerland). Control cultures containing either complete cell culture medium alone or pokeweed mitogen (PWM, 10 μg/ml; Sigma, UK) were set up in parallel. PBMC were cultured at 37 ^o^C in the presence of 5% CO_2_ for 18–21 hours followed by a further 4 hours in the presence of Brefeldin-A (Sigma).

### Flow cytometry

All labelling was performed at 4 °C. Cultured PBMC were washed and labelled for 15 min with LIVE/DEAD fixable violet dead cell stain (Invitrogen, UK). Subsequently, cells were washed and surface labelled for 20 min. After a further wash cells labelled with unconjugated antibodies were incubated with a fluorochrome-labelled secondary antibody for 20 min. Before intracellular labelling (ICS), cells were washed and fixed with 1% formaldehyde (Cytofix, BD Biosciences, UK) over night at 4 °C, treated with Perm/Wash (BD Biosciences, UK) buffer according to manufacturer’s instruction and intracellularly labelled with recombinant bivalent boIL-22 Fab antibody, polyclonal rabbit anti-boIL-17A-biotin (Kingfisher Biotech, USA) and anti-boIFN-γ-PE (clone CC302, Bio-Rad AbD Serotec, UK) individually or in combination for 20 min followed by secondary labelling with polyclonal goat anti-huIgG F(ab’)_2_-FITC (Bio-Rad AbD Serotec, UK) and/or Streptavidin-fluorochrome for 20 min. The same labelling strategy was used for all antibody panels, which are summarised in Table 1. As control for stimulation of the individual cell populations, cells were surface labelled as described before and intracellularly labelled with anti-boIFN-γ-PE only (Supplemental Fig. 1). All antibodies were purchased from Bio-Rad AbD Serotec except where stated. Antibodies were pre-titrated to determine optimal working concentrations (data not shown). Samples were acquired with a CyAn ADP analyser equipped with 405, 488 and 642 nm lasers (Beckman Coulter, USA) using Summit software version 4.3.02. More than 100,000 singlet live lymphocytes were acquired. Un-labelled, single stained cells and FMO controls were used as controls and to calculate compensation. Data analysis was performed using FlowJo version 10.0.7 software (TreeStar, USA).

### Statistical analysis

Statistical analysis was performed using GraphPad Prism, version 6.04 (GraphPad Software, USA). The D’Agostino & Pearson omnibus normality test was used to assess Gaussian distribution, and parametric or non-parametric tests used where appropriate.

## Additional Information

**How to cite this article**: Steinbach, S. *et al*. CD4+ and γδ T Cells are the main Producers of IL-22 and IL-17A in Lymphocytes from *Mycobacterium bovis*-infected Cattle. *Sci. Rep.* **6**, 29990; doi: 10.1038/srep29990 (2016).

## Supplementary Material

Supplementary Information

## Figures and Tables

**Table 1:** Summary of flow cytometry antibody labelling panels^a^.

Procedure	Cell surface	Intracellular
Screening of boil-22 antibody clones	1^°^: anti-boCD4-AF647 (CC8, Bio-Rad AbD Serotec)	1^°^: recombinant boil-22 Fab antibody (Bio-Rad AbD Serotec)2^°^: polyclonal goat anti-huIgG F(ab’)_2_-FITC (Bio-Rad AbD Serotec)
CD4+ T cell population	1^°^: anti-boCD4-AF647	1^°^: boil-22 Fab antibody (clone 58, Bio-Rad AbD Serotec), polyclonal rabbit anti-boIL-17A-biotin (Kingfisher Biotech)2^°^: anti-huIgG F(ab’)_2_-FITC, Streptavidin-PE (BD Pharmingen)
CD8+ T cellpopulation	1^°^: anti-boCD8 (CC63, Bio-Rad AbD Serotec)2^°^: polyclonal goat anti-muIgG2a-PE (Invitrogen)	1^°^: boil-22 Fab, anti-boIL-17A-biotin2^°^: anti-huIgG F(ab’)_2_-FITC, Streptavidin-AF700 (Invitrogen)
NK cell population	1^°^: anti-boCD335 (NKp46, AbD Serotec)2^°^: polyclonal goat anti-muIgG1-PE (Invitrogen)	1^°^: anti-boIL-22 Fab, anti-boIL-17A-biotin2^°^: anti-huIgG F(ab’)_2_-FITC, Streptavidin-AF700
γδ T cell population	1^°^: anti-boγδ TCR (GB21A, Kingfisher Biotech)2^°^: polyclonal goat anti-muIgG2b-AF633 (Invitrogen)	1^°^: anti-boIL-22 Fab, anti-boIL-17A-biotin2^°^: anti-huIgG F(ab’)_2_-FITC, Streptavidin-PE
IL-22/IL-17A co-producers	1^°^: anti-boγδ TCR and anti-boCD4-AF6472^°^: polyclonal goat anti-IgG2b-PE-Cy7 (SouthernBiotech)	1^°^: anti-boIL-22 Fab, anti-boIL-17A-biotin and anti-bo IFN-γ-PE (CC302, Bio-Rad AbD Serotec)2^°^: anti-huIgG F(ab’)_2_-FITC, Streptavidin-AF700

^a^Antibodies are available from the commercial companies listed in the table.
