# Supplementary material for: CD4+ and γδ T Cells are the main Producers of IL-22 and IL-17A in Lymphocytes from Mycobacterium bovis-infected Cattle
Source: Sci Rep. 2016 Jul 18;6:29990. doi: 10.1038/srep29990 (PMC4947955; doi:10.1038/srep29990)
Supplement: Supplementary Information [file srep29990-s1.pdf]

Supplementary Information

Manuscript: CD4+ and γδ T Cells are the main Producers of IL-22 and IL-17A in Lymphocytes from *Mycobacterium bovis*-infected Cattle.

Authors and Affiliation: Sabine Steinbach, H. Martin Vordermeier, Gareth J. Jones*
TB Immunology and Vaccinology, Department of Bacteriology, Animal and Plant Health Agency, New Haw, Addlestone, Surrey, KT15 3NB, United Kingdom

Supplemental figure S1.

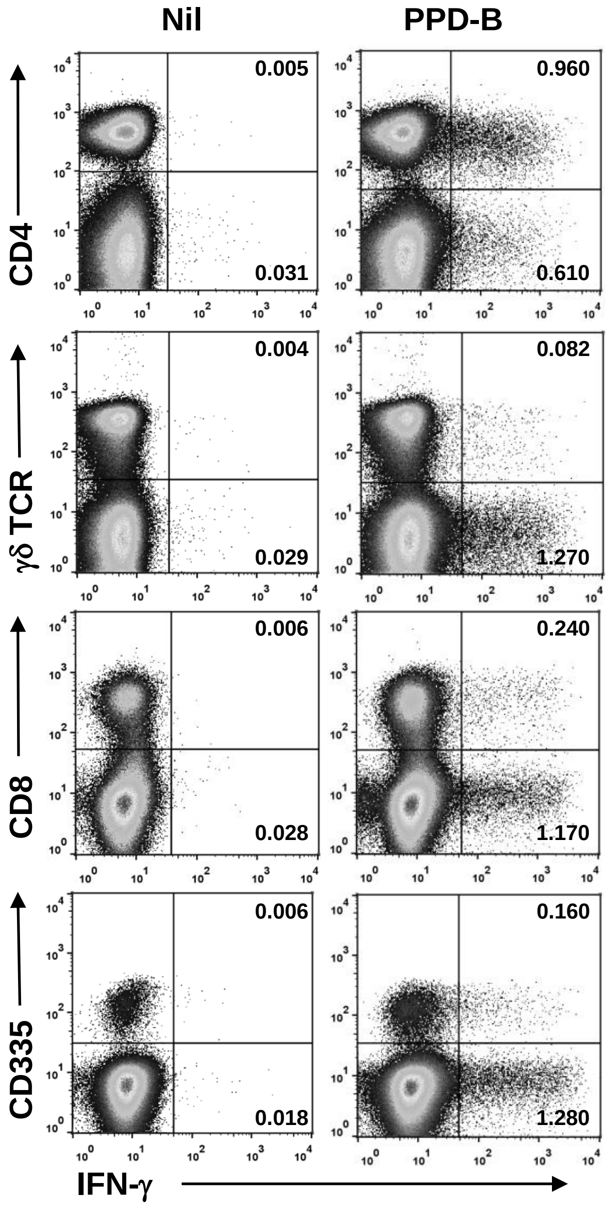

Supplemental figure S1. *M. bovis* specific induction of IFN-γ production in multiple cell populations. PBMC from a TB-reactor animal were stimulated with PPDB and flow cytometry analysis performed to enumerate IFN-γ production in different lymphocyte populations. Numbers represent the percentage of single, live bovine lymphocytes.

Supplemental figure S2.

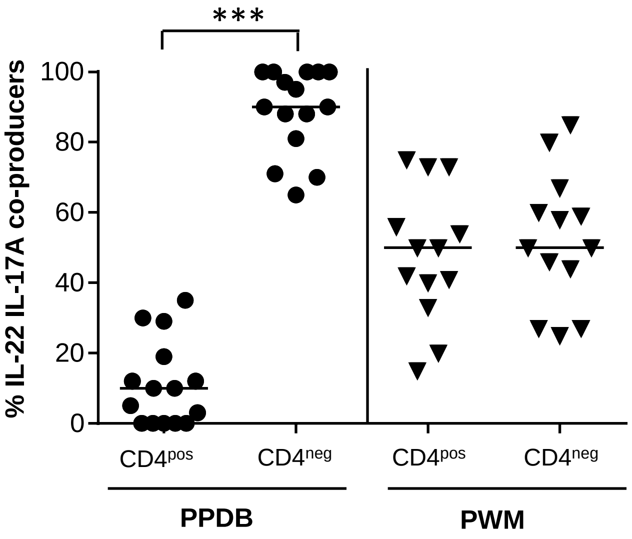

Supplemental figure S2. ***M. bovis*** **antigens induce IL-22 / IL-17A co-producers mainly in the CD4**$^{\mathbf{neg}}$ **lymphocyte compartment.** PBMC from a TB-reactor animal were stimulated with PPDB or PWM and flow cytometry analysis performed to enumerate simultaneous production of IL-22 and IL-17A in CD4^{pos} and CD4^{neg} lymphocyte compartments. The results are presented as the relative proportion of IL-22 IL-17A co-producers that label with or without CD4. Each symbol represents an individual animal while horizontal lines represent the median value. *** $p<0.001$, Paired Students T test.
